# The efficacy and safety of cuttlebone for lowering serum phosphate in patients with end-stage renal disease: a meta-analysis of randomized controlled trials

**DOI:** 10.3389/fphar.2023.1206366

**Published:** 2023-07-24

**Authors:** Hsiao-Tien Chen, Kuo-Chuan Hung, Chin-Wei Hsu, Jui-Yi Chen, Chien-Cheng Liu, I-Wen Chen, Cheuk-Kwan Sun

**Affiliations:** ^1^ Department of Chinese Medicine, Chi Mei Medical Center, Tainan City, Taiwan; ^2^ School of Medicine, College of Medicine, National Sun Yat-sen University, Kaohsiung City, Taiwan; ^3^ Department of Anesthesiology, Chi Mei Medical Center, Tainan City, Taiwan; ^4^ Department of Pharmacy, Chi Mei Medical Center, Tainan City, Taiwan; ^5^ School of Pharmacy, Kaohsiung Medical University, Kaohsiung City, Taiwan; ^6^ Division of Nephrology, Department of Internal Medicine, Chi Mei Medical Center, Tainan City, Taiwan; ^7^ Department of Health and Nutrition, Chia Nan University of Pharmacy and Science, Tainan City, Taiwan; ^8^ Department of Anesthesiology, E-Da Hospital, I-Shou University, Kaohsiung City, Taiwan; ^9^ Department of Anesthesiology, Chi Mei Medical Center, Liouying, Tainan City, Taiwan; ^10^ Department of Emergency Medicine, E-Da Dachang Hospital, I-Shou University, Kaohsiung City, Taiwan; ^11^ School of Medicine for International Students, College of Medicine, I-Shou University, Kaohsiung City, Taiwan

**Keywords:** cuttlebone, serum phosphate, meta-analysis, end-stage renal disease, phosphate-binding agents

## Abstract

**Background:** The efficacy of cuttlebone for treating hyperphosphatemia in patients with end-stage renal disease and its safety remained unclear.

**Methods:** Randomized controlled trials comparing the efficacy of cuttlebone with conventional interventions were retrieved from MEDLINE, EMBASE, Cochrane Library, Airiti Library, and other major Chinese databases until 1 February 2023. The primary outcome was circulating phosphate concentration, while secondary outcomes included circulating calcium and intact parathyroid hormone levels, calcium–phosphorus product, and treatment-related side-effects.

**Results:** Analysis of nine studies published between 2000 and 2019 including 726 participants showed a lower circulating phosphate concentration in the cuttlebone group than in controls [mean difference (MD) = −0.23, 95% CI: −0.39 to −0.06, *p* = 0.006, I^2^ = 94%, 726 patients] and a dose-dependent effect of cuttlebone against hyperphosphatemia. Therapeutic benefits were noted after both short-term (1–2 months) and long-term (3–6 months) treatments. Besides, patients receiving hemodialysis showed a better response to cuttlebone than those receiving peritoneal dialysis. There was no difference in circulating calcium level (mean difference = 0.03, 95% CI: −0.01 to 0.07, *p* = 0.17, I^2^ = 34%, 654 patients), while patients receiving cuttlebone showed lower circulating iPTH level and calcium-phosphorus product (MD = −43.63, 95% CI: −74.1 to −13.16, *p* = 0.005, I^2^ = 76%, 654 patients), (MD = −0.38, 95% CI: −0.38 to −0.01, *p* = 0.04, I^2^ = 83%, 520 patients). No difference in the risks of constipation, gastrointestinal discomfort, and elevated blood calcium was noted between the two groups.

**Conclusion:** Compared with conventional phosphate-binding agents, cuttlebone more efficiently suppressed hyperphosphatemia with a dose-dependent effect. The limited number of included studies warrants further clinical investigations to verify our findings.

**Systematic Review Registration:** https://www.crd.york.ac.uk/prospero/, identifier CRD42023396300.

## 1 Introduction

Hyperphosphatemia is the most common complication of end-stage renal disease (ESRD) and is significantly associated with the severity of renal dysfunction (Shaman and Kowalski, 2016; Isakova et al., 2017). Growing evidence suggests that hyperphosphatemia, calcium-phosphorus product, and hyperparathyroidism may increase the risk of chronic kidney disease-mineral bone disease (CKD-MBD), fractures, and progression of kidney disease (Holden et al., 2020; Pazianas and Miller, 2021). Previous studies have shown that managing blood phosphorus concentration is more effective for reducing the incidence of cardiovascular disease and mortality rate than controlling the levels of circulating calcium and parathyroid hormone (PTH) (Vervloet and van Ballegooijen, 2018; Doshi and Wish, 2022). Currently, the management strategies for hyperphosphatemia include adequate dialysis, a strict restriction of phosphorus diet, and the use of phosphorus-binding agents. During hemodialysis, the net positive phosphorus balance is in the range of 1,200–1,400 milligrams per day, while each dialysis session can only remove 500–600 milligrams of phosphorus (Li et al., 2019). Therefore, even with the strictest restriction of daily dietary phosphorus (i.e., 1,000 milligrams), a patient undergoing dialysis would still have approximately 600 milligrams of phosphorus being retained in the body (Waheed et al., 2013; Li et al., 2019; Naber and Purohit, 2021). At present, phosphorus-lowering drugs are divided into calcium carbonate/acetate phosphate binders (CBPBs) and non-calcium-based phosphate binders (NCBPBs), which are known to cause hypercalcemia and the development of metastatic calcification (Tsai et al., 2020; Ogata et al., 2021). In addition to gastrointestinal adverse effects common to both agents, each of them has its specific concerns in clinical use. While CBPBs are known to elevate the risk of cardiovascular complications in patients on dialysis, NCBPBs are more expensive and related to an increased risk of metal deposition (e.g., lanthanum and sevelamer) (Sprague, 2007; Habbous et al., 2017). Therefore, identification of a suitable phosphorus reduction regimen is still an important topic in current medical research.

Cuttlebone, also known as squid bone, is the dried inner shell of *Sepiella maindroni de Rochebrune, Sepia subaculeata Sasaki,* or *Sepia esculenta Hoyle*, family of *Squididae* (Palaveniene et al., 2018). In ancient Asia, cuttlebone is a traditional Chinese medicine used as an antacid and is believed to be effective against a variety of gastric diseases (Chien et al., 2015). Having many superimposed spaces with complex calcified columns and organic membranes (Guo, 2008), cuttlebone consists a mixture of calcium carbonate (87.3%–91.8%) (Tangsuksant et al., 2023) and small amounts of organic matter (3%–4.5%), β-chitin and proteins. Prior *in vitro* experimental studies have demonstrated that chitin from cuttlebone could enhance wound healing through stimulating macrophages, inducing nitric oxide production in macrophages, increasing the expression of pro-inflammatory cytokines, and enhancing fibroblast migration (Lee et al., 2013; Lim et al., 2015). Cuttlebone, which is primarily composed of calcium carbonate, may help lower high blood phosphate levels due to its high calcium content (Liao and Xiong, 2019). A previous review reported the effectiveness of cuttlebone for reducing serum phosphorus levels without affecting serum calcium concentration (Meng and Fu, 2015), which may be explained by the large size of calcium particles in cuttlebone that was experimentally shown to be poorly absorbed by the body following oral intake (Chien et al., 2015). The phosphate-lowering effect of cuttlebone may also be attributed to other constituents in cuttlebone such as Mg and Fe, which has been shown to block phosphorus iron absorption (Chien et al., 2015; Palaveniene et al., 2019; Palaveniene et al., 2020). The aim of this meta-analysis was to investigate the efficacy and safety of using cuttlebone for reducing circulating phosphate levels in patients with end-stage renal disease.

## 2 Materials and methods

This meta-analysis was pre-registered in the PROSPERO (CRD42023396300), with reporting of results based on the preferred reporting items for systematic reviews and meta-analysis (PRISMA) statement. Supplementary Figure S1 presented the flowchart that illustrated the step-by-step methodology employed in the current meta-analysis.

### 2.1 Search strategy and databases

We systematically searched randomized controlled trials (RCTs) that evaluated the efficacy of cuttlebone for reducing serum phosphate. Eight databases including Medline, Embase, Cochrane library, Google Scholar, China National Knowledge Infrastructure, VIP Database for Chinese Technical Periodicals, Chinese Biomedical Literature, and Wanfang Database were searched from inception to 1 February 2023 to identify relevant articles without limits on publication year, sample size, country, and language. All literature search was performed by two independent reviewers. We combined the medical subject headings (e.g., MeSH terms in Medline) and free texts to enhance the efficiency of our search using the following terms: (“cuttlebone” or “Hai-Piao-Xiao” or “cuttlefish bone” or “Sepiella maindroni de Rochebrune” or “Octopus japonicus” or “Sepiae Endoconcha”) and (“renal failure” or “end stage renal disease” or “dialysis” or “hemodialysis” or “peritoneal dialysis” or “kidney failure” or “renal insufficiency”) and (“hyperphosphatemia” or “Serum phosphate” or “phosphorus”). Supplemental Table S1 summarized the search strategy for one of the databases [i.e., Medline (OVID)]. Two reviewers independently screened the titles/abstracts and full-texts of the eligible articles. Besides, the reference lists of the selected articles and review articles were manually inspected to find potentially missing articles. Disagreements on study inclusion were discussed between the two reviewers and resolved by consulting a third reviewer.

### 2.2 Inclusion and exclusion criteria

Studies were considered eligible if they met all of the following criteria: (1) Population: adults (i.e., ≥18 years): with chronic kidney disease based on the definition of individual studies or those with renal failure undergoing hemodialysis or peritoneal dialysis; (2) Intervention: the use of cuttlebone as an intervention strategy regardless of dosage and treatment duration; (3) Control: the use of active control or placebo for comparison; (4) Outcomes: the change in serum phosphate concentration, serum levels of calcium, intact parathyroid hormone (iPTH), and serum calcium-phosphorus product, as well as treatment-related side effects. Published RCTs including full-length articles and conference abstracts were considered eligible. Articles that were (1) focused on the pediatric population, (2) did not provide information on outcomes, or (3) those presented as review articles, case reports, and case series were excluded.

### 2.3 Primary outcomes and data extraction

The primary endpoint was the change in serum phosphate concentration, while the secondary outcomes included the changes in serum levels of calcium, iPTH, and serum calcium-phosphorus product, as well as treatment-related side effects. Subgroup analyses were performed based on the dosage of cuttlebone, duration of treatment, type of dialysis (i.e., hemodialysis vs. peritoneal dialysis), type of control group (calcium-based vs. non-calcium-based regimens). If a study provided data on treatment outcomes at different time points, we used the data with the longest follow-up to analyze the overall size effect. For subgroup analyses on dosage and duration of treatment, data from cuttlebone of different dosages [i.e., marked as study (D1) and study (D2)] or treatment durations [i.e., marked as study (T1) and study (T2)] from a study were extracted.

Data extraction was performed by two independent reviewers to collect first author name, study design, publication year, number of patients, therapeutic duration and dosage of cuttlebone, the type of control group, duration of dialysis, follow-up period, side effects (e.g., gastrointestinal discomfort), and country. For studies that did not provide adequate data for analysis, we contacted the corresponding authors to request the missing information. In case of disagreements on data collection, a third reviewer was consulted for arbitration.

### 2.4 Quality of studies

The risk of bias was independently investigated by two experienced reviewers based on the Cochrane Collaboration’s risk of bias tools (ROB 2.0). The risk of bias was categorized into “low”, “high”, and “some concerns” based on the following domains: randomization process, deviations from the intended interventions, missing outcome data, measurement of the outcome, selection of the reported result, and overall risk of bias. Any discrepancies in the assessment of quality of studies were resolved through a consensus.

### 2.5 Statistical analyses

In this study, all statistical analyses were conducted with the assistance of the Cochrane Review Manager tool. For conducting primary analysis, a random-effects model was used to calculate the overall effect size. Dichotomous and continuous data were displayed as risk ratios (RRs) and mean difference (MD), respectively. Additionally, 95% confidence intervals (95% CIs) are provided, and the presence of heterogeneity was evaluated using I^2^ statistics, where I^2^>50% was regarded as significant. To ensure the reliability of the available evidence, a leave-one-out sensitivity analysis was performed. This analysis helped to determine how much of an impact each individual study had on the overall results. The possibility of publication bias was examined by visual inspection of the funnel plot for the observed outcomes when there was a minimum of 10 trials with a shared outcome. A result was considered statistically significant if the probability value (*p*) was less than 0.05.

## 3 Results

### 3.1 Literature search

**FIGURE 1:**
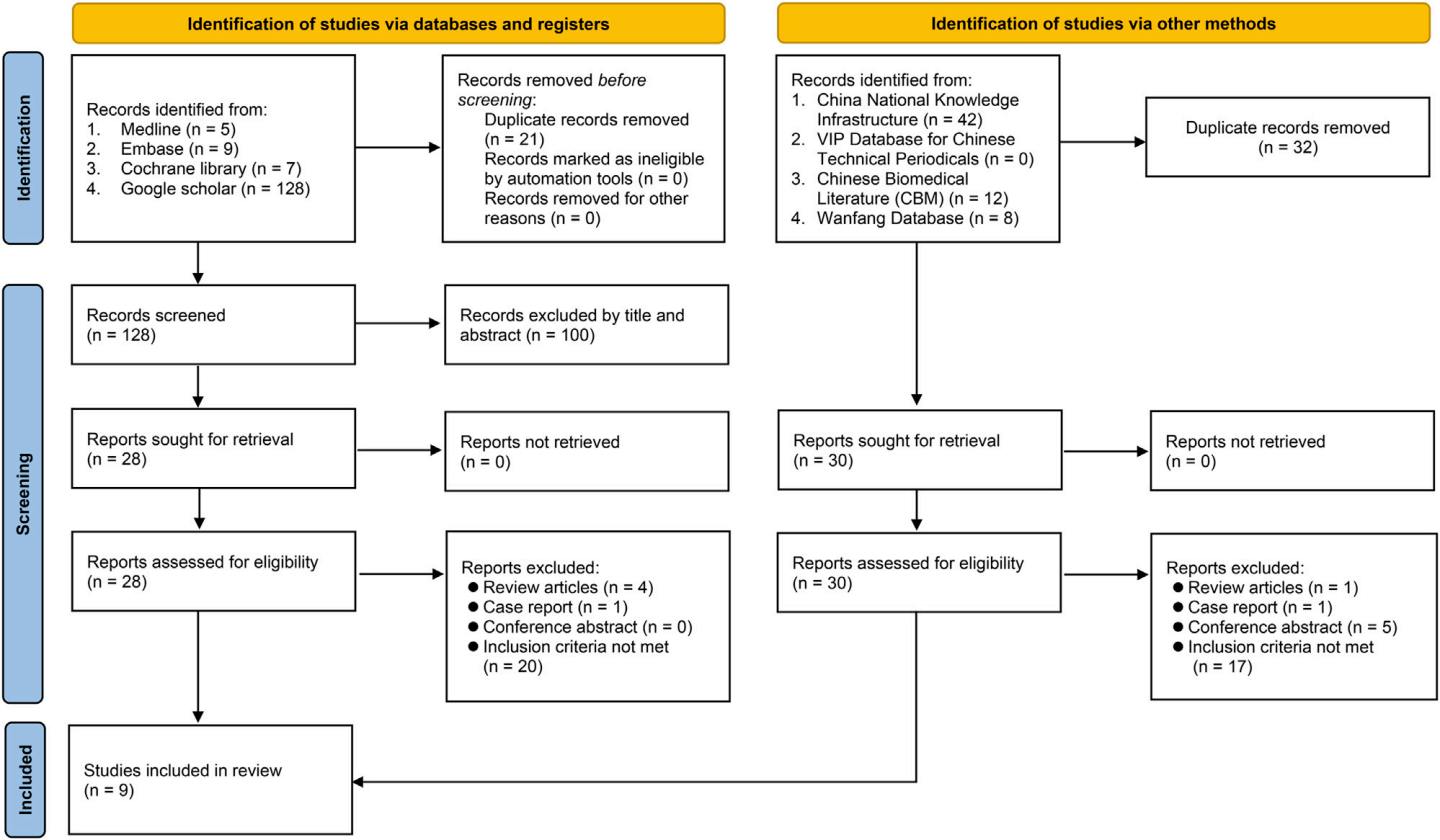
Flowchart of study selection.

Initial search on databases including Medline, Embase, Cochrane library, and Google scholar identified 149 records, of which 121 were excluded because they were duplicated or did not meet the inclusion criteria based on the title/abstract screening. Of the 28 articles retrieved and reviewed in full text, three were deemed eligible. In addition to the six articles further identified from other databases (e.g., Wanfang database), nine studies published between 2000 and 2019 were finally included in the present meta-analysis (Liu, 2000; Liu and Hu, 2005; Guo, 2008; Lee et al., 2012; Zhu and Li, 2013; Liu and An, 2014; Tang et al., 2016; Cheng et al., 2018; Liao and Xiong, 2019). The process of database search is shown in Figure 1.

### 3.2 Characteristics and quality of studies

The nine eligible studies involved a total of 726 adults, of whom 390 were in the cuttlebone group and 336 were in the control group (i.e., active control or placebo) (Table 1). For study design, two studies were three-arm trials (Liu and Hu, 2005; Zhu and Li, 2013), while seven studies were two-arm trials (Liu, 2000; Guo, 2008; Lee et al., 2012; Liu and An, 2014; Tang et al., 2016; Cheng et al., 2018; Liao and Xiong, 2019). The follow-up duration of the nine included studies was between one and 6 months. Two trials provided treatment outcomes at two or more time points (Guo, 2008; Tang et al., 2016); while one study analyzed the outcomes after three and 6 months of treatment (Guo, 2008), the other gave available data following treatment for one, two, and 3 months (Tang et al., 2016). The number of patients in each RCT ranged from 60 to 120. Six studies included patients with ESRD receiving hemodialysis (range: 1–23 months) (Liu and Hu, 2005; Lee et al., 2012; Zhu and Li, 2013; Liu and An, 2014; Tang et al., 2016; Liao and Xiong, 2019), while two involved participants with ESRD undergoing peritoneal dialysis for more than 3 months (Guo, 2008; Cheng et al., 2018). One study focused on patients with advanced renal failure without specifying the type of dialysis therapy (Liu, 2000). For the intervention group, cuttlebone was prescribed as a monotherapy (dosage: 1–5 g thrice a day) in eight RCTs (Liu, 2000; Liu and Hu, 2005; Guo, 2008; Lee et al., 2012; Liu and An, 2014; Tang et al., 2016; Cheng et al., 2018; Liao and Xiong, 2019), while one three-arm RCT used cuttlebone alone or cuttlebone combined with sevelamer hydrochloride as the intervention strategy (Zhu and Li, 2013). For the control group, the therapeutic regimens included lanthanum carbonate, CaCO_3_, charcoal, and sevelamer hydrochloride (i.e., active control), while three studies did not specify this information (Liu, 2000; Guo, 2008; Liu and An, 2014). Supplementary Table S2 summarized the unique side-effects associated with cuttlebone, namely constipation, gastrointestinal discomfort, and hypercalcemia.

**TABLE 1:** Characteristics of studies (*n* = 9).

Author (year)	Age (years) (I vs. C)	N (I vs. C)	Patients (define of hyperphosphatemia)	Duration of dialysis	Intervention and control group (dosage/frequency)	Outcomes	Follow-up
Cheng (2018)	53.8 vs. 57.0	60 vs. 60	CAPD with hyperphosphatemia (≥1.78 mmol/L)	>3 months	I: Cuttlebone (1g/TID) C: lanthanum carbonate (500mg/TID)	a, b, c, d	3 months
Guo (2008)	53.4 vs. 52.2	73 vs. 35	CAPD with hyperphosphatemia (≥1.60 mmol/L)	>3 months	I: Cuttlebone (1g/TID)	a, b, c, d	T1:3 months
					C: NA		T2: 6 months
Lee (2012)	45 vs. 45	30 vs. 30	Hemodialysis with hyperphosphatemia (≥1.78 mmol/L)	23 months	I: Cuttlebone (1g/TID)	a, b, c, d	2 months
					C: CaCO3 (0.3g/TID)		
Liao (2019)	51.5 vs. 51.5	32 vs. 32	hemodialysis with hyperphosphatemia (≥1.78 mmol/L)	>3 months	I: Cuttlebone (1g/TID)	a, b, c, d	6 months
					C: CaCO3 (0.75g/TID)		
Liu (2000)	24–70 vs.26-62	40 vs. 32	Renal failure with hyperphosphatemia (NA)	NA	I: Cuttlebone (5g/TID)	a	1 month
					C: NA		
Liu (2005)	58 vs. 58	25 vs. 49	Hemodialysis with hyperphosphatemia (≥1.78 mmol/L)	18 months	I:Cuttlebone (3g/TID)	a, b, c	3 months
					C1: CaCO3 (1.5g/TID)		
					C2: routine care		
Liu (2014)	51.5 vs. 49.3	30 vs. 30	Hemodialysis with hyperphosphatemia (≥1.78 mmol/L)	>3 months	I: Cuttlebone (2g/TID)	a, b, c	6 months
					C: NA		
Tang (2016)	62.5 vs. 59.2	40 vs. 38	Hemodialysis with hyperphosphatemia (≥1.78 mmol/L)	>8 months	I: Cuttlebone (1.7g/TID)	a, b, c, d	T1: 1 month
					C: Charcoal (3 tab/0.3g/TID)		T2: 2 months
							T3: 3 months
Zhu (2013)	50.3 vs. 50.4	60 vs. 30	Hemodialysis with hyperphosphatemia (≥1.78 mmol/L)	>1 month	D1:Cuttlebone (1.5g/TID)	a, b, c, d	2 months
					D2: Cuttlebone (1g/TID) and Sevelamer Hydrochloride		
					(800–1600mg/TID)		
					C: Sevelamer Hydrochloride (800–1600mg/TID)		

iPTH, intact parathyroid hormone; I, intervention group; C, control group; CAPD, continuous ambulatory peritoneal dialysis; a, Serum phosphorus; b, Serum calcium; c, iPTH; d, calcium-phosphorus product.

**FIGURE 2:**
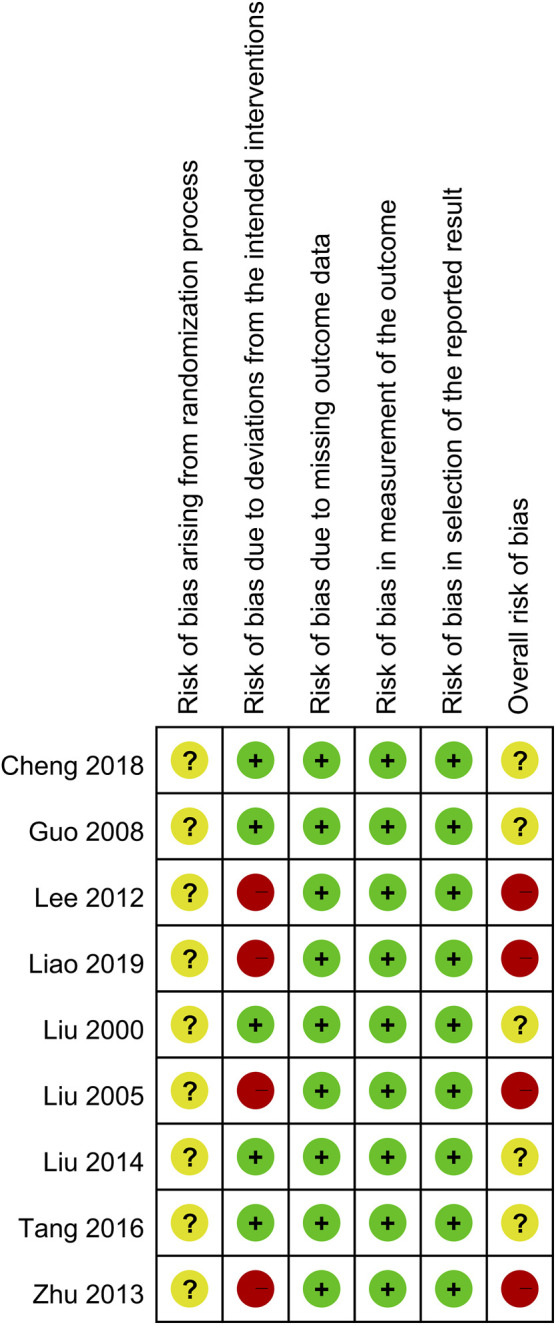
Risk of bias summary of individual studies based on authors’ judgements.

Regarding the process of randomization, all studies did not report allocation concealment; therefore, the risk of bias on this domain was considered to be of “some concern”. Four studies, which did not specify the restriction of dietary and medication use that may potentially influence the outcomes between treatment and control groups (Liu and Hu, 2005; Lee et al., 2012; Zhu and Li, 2013; Liao and Xiong, 2019), were deemed at high risk of deviations from the intended interventions. In summary, the overall risk of bias was regarded as “some concern” and “high risk of bias” in five (Liu, 2000; Guo, 2008; Liu and An, 2014; Tang et al., 2016; Cheng et al., 2018) and four (Liu and Hu, 2005; Lee et al., 2012; Zhu and Li, 2013; Liao and Xiong, 2019) studies, respectively (Figure 2).

### 3.3 Outcomes

#### 3.3.1 Primary outcome: impact of cuttlebone on circulating phosphate level

**FIGURE 3:**
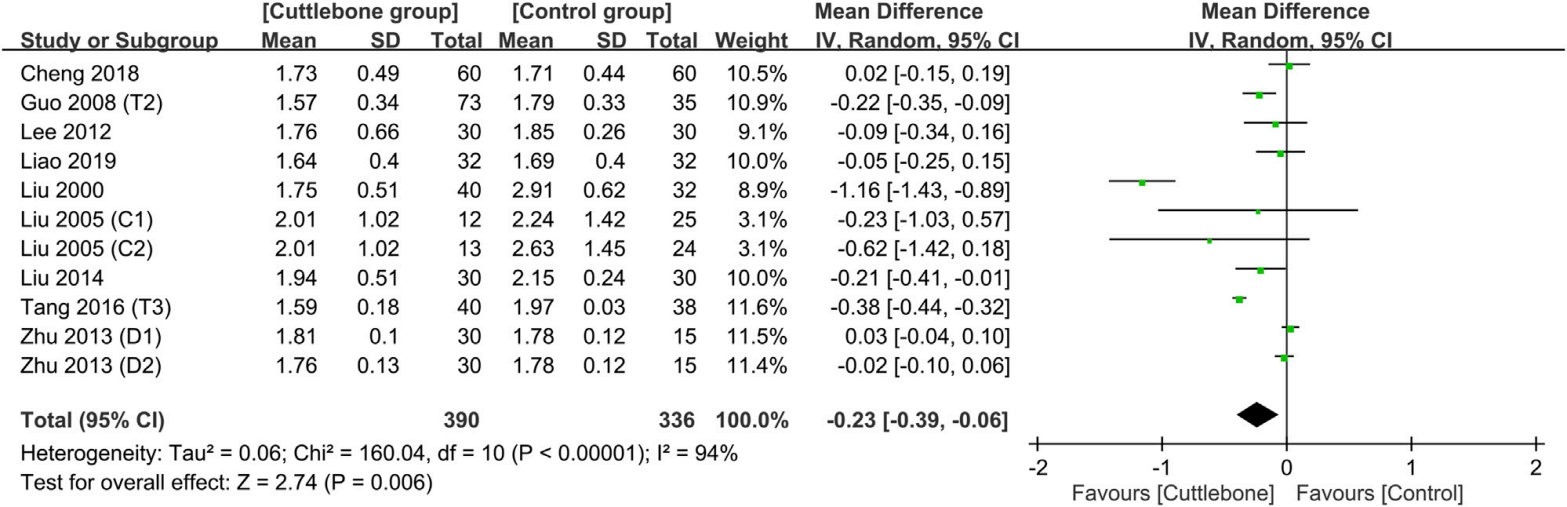
Forest plot comparing circulating phosphate concentrations between cuttlebone and control groups. CI, confidence interval; IV, inverse variance; SD, standard deviation.

Meta-analysis revealed a lower circulating phosphate concentration in patients receiving cuttlebone compared to that in the control group (MD = −0.23, 95% CI: -0.39 to −0.06, *p* = 0.006, I^2^ = 94%, 726 patients) (Figure 3) (Liu, 2000; Liu and Hu, 2005; Guo, 2008; Lee et al., 2012; Zhu and Li, 2013; Liu and An, 2014; Tang et al., 2016; Cheng et al., 2018; Liao and Xiong, 2019). However, the statistical significance disappeared on sensitivity analysis when one study (Liu, 2000) that adopted a large dose of cuttlebone (i.e., 5 g TID) was removed (MD = −0.13, 95% CI: -0.28 to 0.01, *p* = 0.07, I^2^ = 92%, 654 patients). Funnel plot showed an overall symmetry, suggesting a low risk of publication bias (Supplementary Figure S2).

**FIGURE 4:**
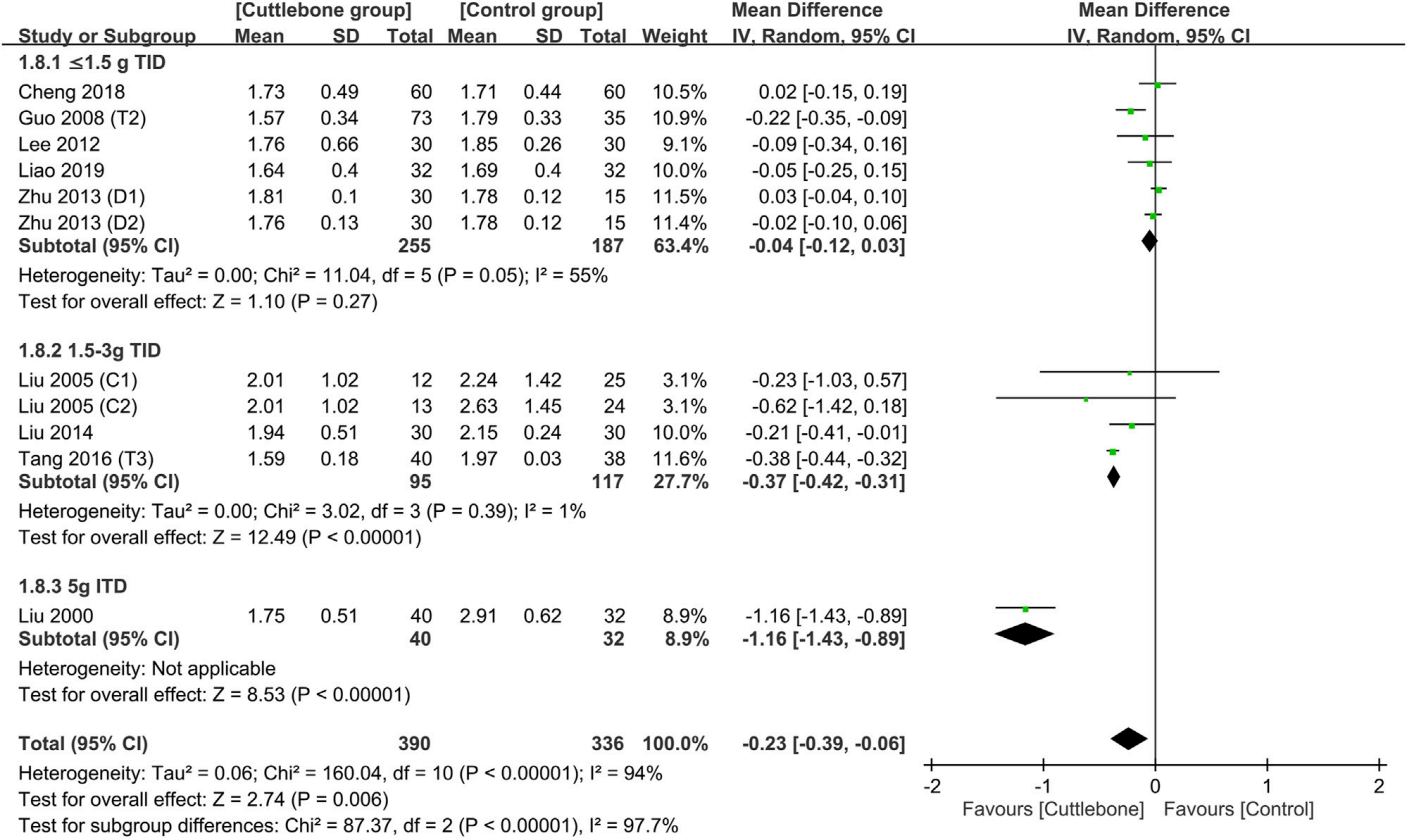
Subgroup analysis showing the difference in circulating phosphate concentrations between cuttlebone and control groups according to cuttlebone dosage. CI, confidence interval; IV, inverse variance; SD, standard deviation.

**FIGURE 5:**
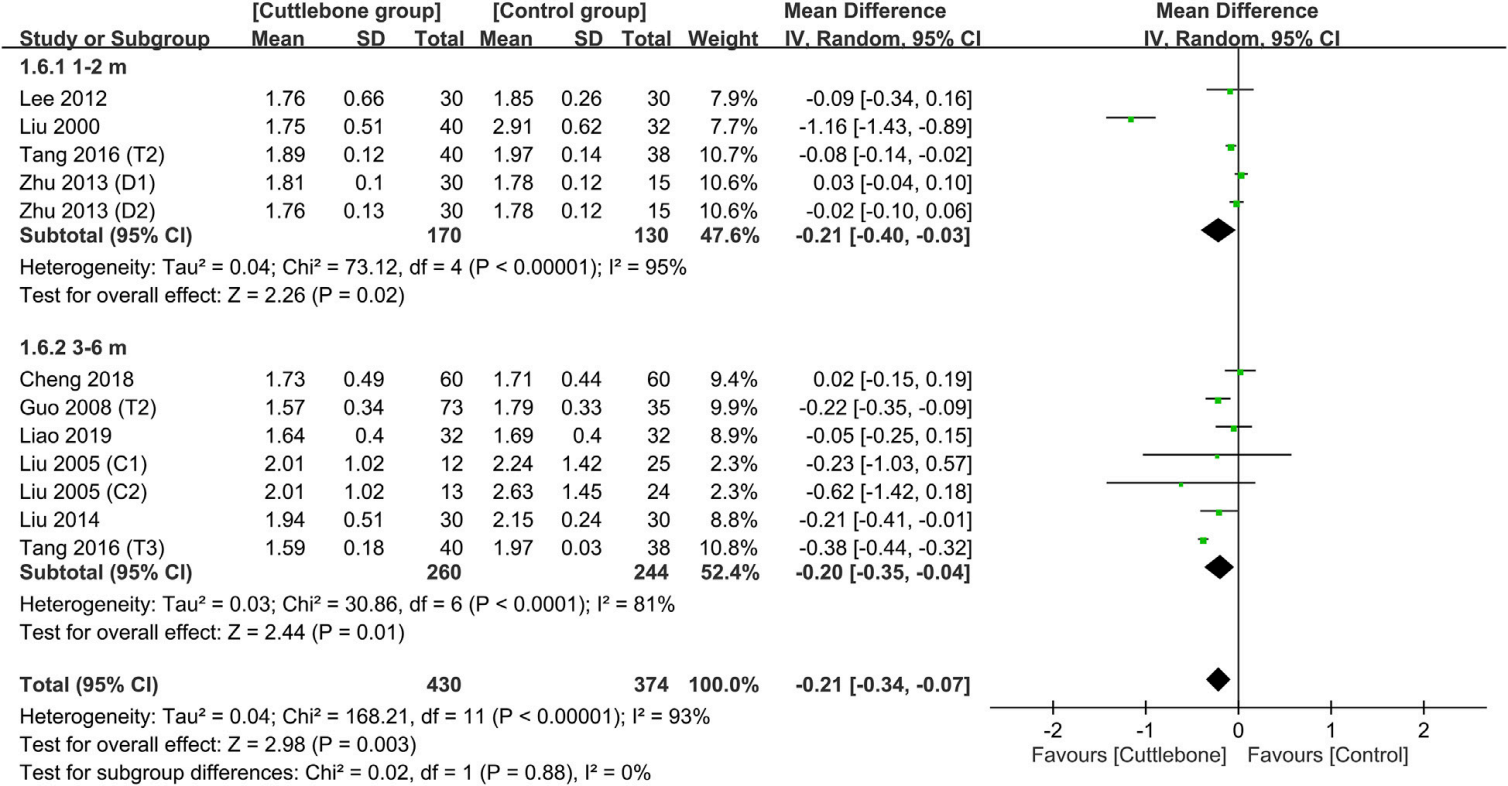
Subgroup analysis demonstrating the difference in circulating phosphate concentrations between cuttlebone and control groups focusing on the duration of treatment. CI, confidence interval; IV, inverse variance; SD, standard deviation.

Subgroup analysis focusing on the dosage of cuttlebone indicated a dose-dependent effect of cuttlebone against hyperphosphatemia. Cuttlebone at a medium-to-large dosage (i.e., 1.5–3 g TID and 5 g TID) was effective for reducing circulating levels of phosphate, while a low dose (i.e., ≤1.5 g) was not beneficial (Figure 4) (Liu, 2000; Liu and Hu, 2005; Guo, 2008; Lee et al., 2012; Zhu and Li, 2013; Liu and An, 2014; Tang et al., 2016; Cheng et al., 2018; Liao and Xiong, 2019). Subgroup analysis of the duration of treatment demonstrated beneficial effects of cuttlebone after both short-term (i.e., 1–2 months) and long-term (3–6 months) administration (Figure 5). Subgroup analysis based on the type of regimens (i.e., calcium-based vs. non-calcium-based) in the control group supported the beneficial effect of cuttlebone on the reduction of circulating phosphate regardless of the type of regimen used (Supplementary Figure S3). Subgroup analysis pinpointing the type of dialysis (i.e., hemodialysis vs. peritoneal dialysis) revealed a favorable response of cuttlebone in patients receiving hemodialysis, but not in those receiving peritoneal dialysis (Supplementary Figure S4).

#### 3.3.2 Secondary outcome: impact of cuttlebone on circulating calcium and iPTH levels, calcium-phosphorus product, and side effects

**FIGURE 6:**
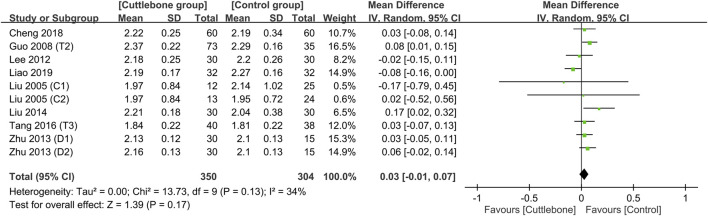
Forest plot comparing circulating calcium concentrations between the cuttlebone and control groups. CI, confidence interval; IV, inverse variance; SD, standard deviation.

Merged results showed no difference in the circulating concentration of calcium between the cuttlebone and control groups (MD = 0.03, 95% CI: -0.01 to 0.07, *p* = 0.17, I^2^ = 34%, 654 patients) (Figure 6) (Liu and Hu, 2005; Guo, 2008; Lee et al., 2012; Zhu and Li, 2013; Liu and An, 2014; Tang et al., 2016; Cheng et al., 2018; Liao and Xiong, 2019). Sensitivity analysis revealed a higher serum calcium in patients with cuttlebone treatment compared to those without when one study (Liao and Xiong, 2019) was removed (MD = 0.05, 95% CI: 0.02 to 0.09, *p* = 0.005, I^2^ = 0%, 590 patients). An overall symmetry on funnel plot inspection indicated a low risk of publication bias regarding the impact of cuttlebone on circulating calcium concentration (Supplementary Figure S5).

**FIGURE 7:**
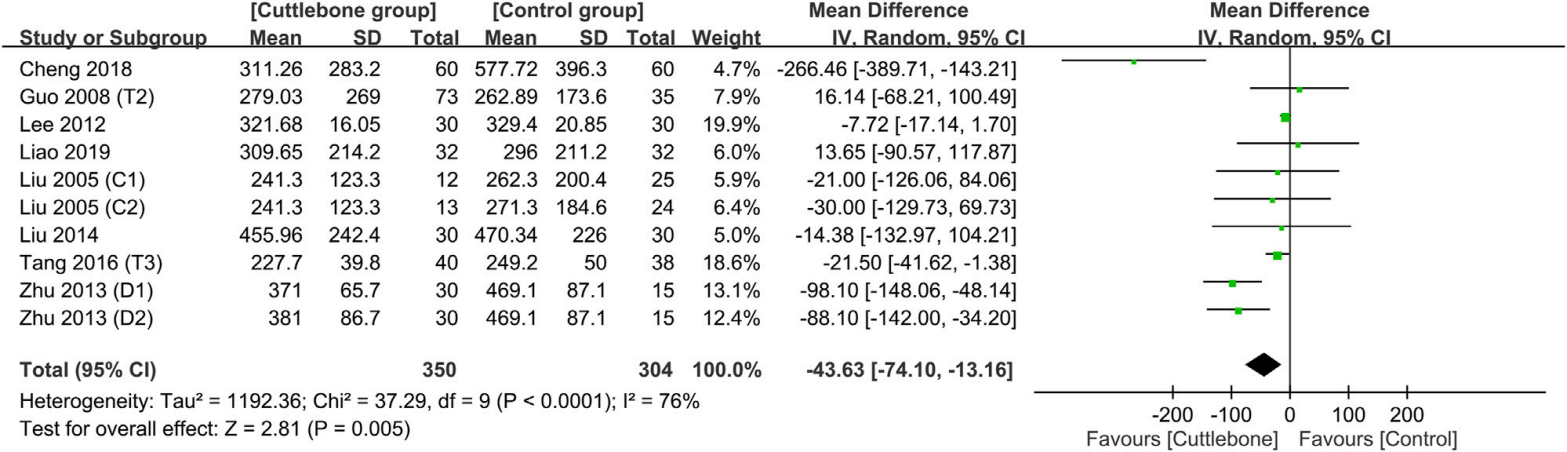
Forest plot showing the difference in circulating intact parathyroid hormone (iPTH) concentrations between the cuttlebone and control groups. CI, confidence interval; IV, inverse variance; SD, standard deviation.

**FIGURE 8:**
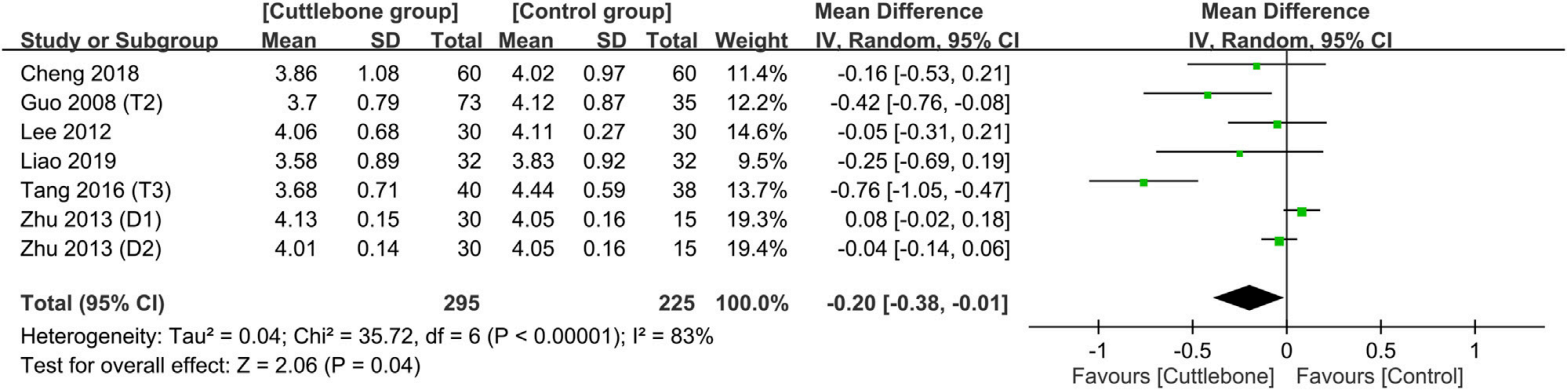
Forest plot demonstrating the difference in calcium-phosphorus product between the cuttlebone and control groups. CI, confidence interval; IV, inverse variance; SD, standard deviation.

Meta-analysis demonstrated a lower circulating iPTH in the cuttlebone group than that in the control group (MD = −43.63, 95% CI: -74.1 to −13.16, *p* = 0.005, I^2^ = 76%, 654 patients, sensitivity analysis: consistent) (Figure 7) (Liu and Hu, 2005; Guo, 2008; Lee et al., 2012; Zhu and Li, 2013; Liu and An, 2014; Tang et al., 2016; Cheng et al., 2018; Liao and Xiong, 2019). Funnel plot implied a low risk of publication bias on this outcome (Supplementary Figure S6). In addition, the use of cuttlebone was associated with a lower calcium-phosphorus product compared to that in the control group (MD = −0.2, 95%CI: -0.38 to −0.01, *p* = 0.04, I^2^ = 83%, sensitivity analysis: inconsistent) (Figure 8) (Guo, 2008; Lee et al., 2012; Zhu and Li, 2013; Tang et al., 2016; Cheng et al., 2018; Liao and Xiong, 2019).

**FIGURE 9:**
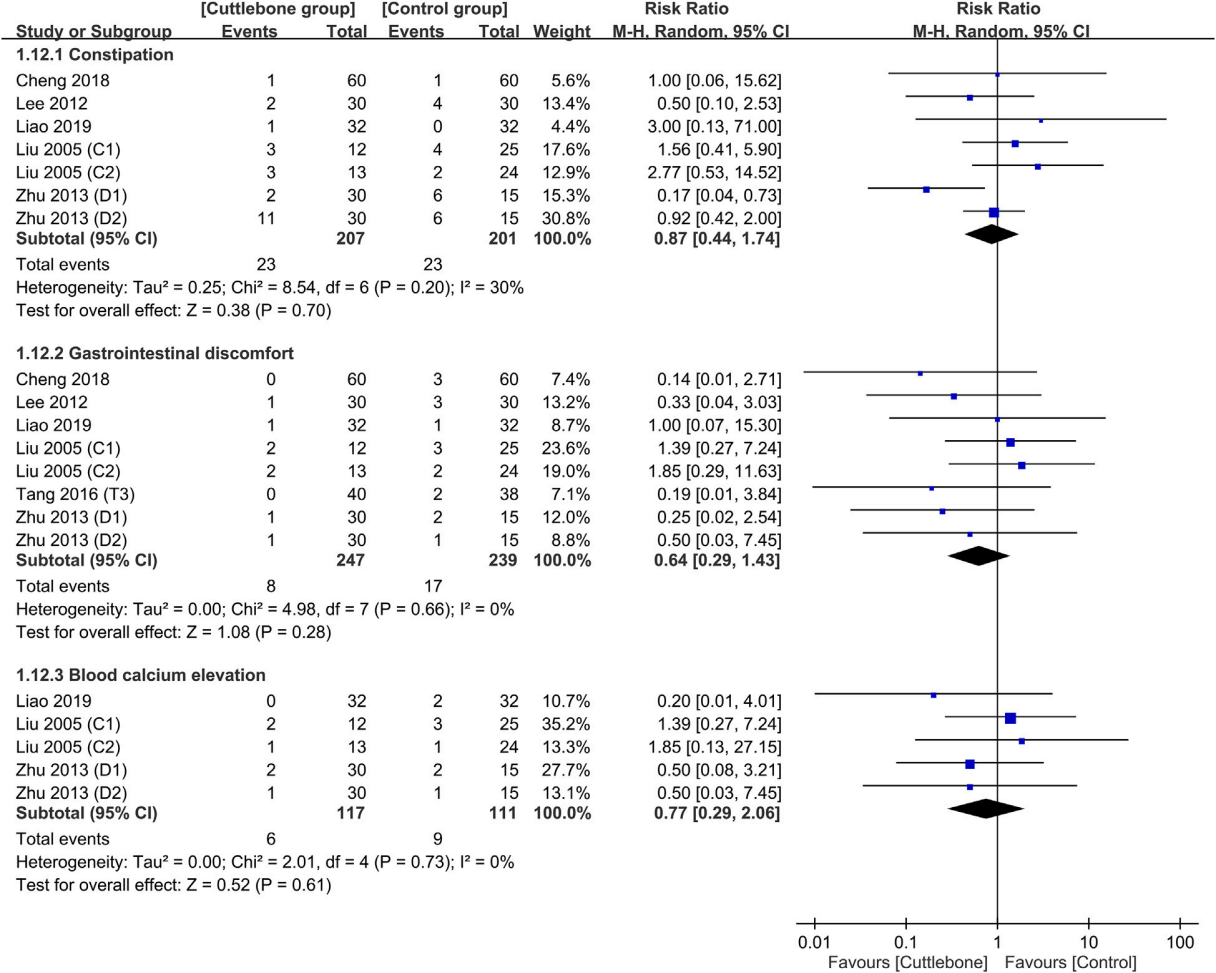
Forest plot comparing the risks of side-effects including constipation, gastrointestinal discomfort, and blood calcium elevation between the cuttlebone and control groups. CI, confidence interval.

A comparison of the safety profile between the cuttlebone and control groups is shown in Figure 9, suggesting no difference in the risk of constipation (RR = 0.87, 95% CI: 0.44 to 1.74, *p* = 0.7, I^2^ = 30%, sensitivity analysis: consistent), gastrointestinal discomfort (RR = 0.64, 95% CI: 0.29 to 1.43, *p* = 0.28, I^2^ = 0%, sensitivity analysis: consistent), and blood calcium elevation (RR = 0.77, 95% CI: 0.29 to 2.06, *p* = 0.61, I^2^ = 0%, sensitivity analysis: consistent) between the two groups.

## 4 Discussion

Hyperphosphatemia contributes to chronic kidney disease-mineral and bone disorder (CKD-MBD) and increases the incidence of cardiovascular disease and mortality rate in individuals with ESRD (Felsenfeld et al., 2015; Zhou et al., 2021). This meta-analysis, combining nine studies and 726 participants, showed an overall lower serum phosphorus, iPTH, and serum calcium-phosphorus product (Ca x Pi) levels in ESRD patients with hyperphosphatemia receiving oral cuttlebone therapy compared to those without treatment. Cuttlebone also exhibited a dose-dependent effect associated with a reduction in circulating phosphate concentration. In addition, compared with calcium carbonate/acetate phosphate binders (CBPBs) and non-calcium-based phosphate binders (NCBPBs), the use of cuttlebone correlated with a lower risk of hyperphosphatemia both after short-term (1–2 months) and long-term (3–6 months) treatments. There was no difference in combined adverse events (i.e., constipation, gastrointestinal discomfort, and elevated blood calcium). Besides, patients receiving hemodialysis showed a better response to cuttlebone compared to those receiving peritoneal dialysis.

The KDIGO (Kidney Disease: Improving Global Outcomes) treatment guidelines recommend adequate dialysis, controlled dietary phosphorus intake, and phosphorus binding agents for patients with CKD Category 5D (i.e., glomerular filtration rate <15 mL/min/1.73 m^2^, kidney failure) (Barreto et al., 2019). Because a neutral phosphate balance cannot be achieved through conventional hemodialysis or peritoneal dialysis alone, more intensive hemodialysis is suggested for improving phosphate control (Zsom et al., 2020). Regarding the restriction of dietary phosphate intake, KDIGO recommended the avoidance of foods with high amounts of phosphate additives and the selection of those with a low phosphorus/protein ratio and a low phosphorus absorption rate (St-Jules et al., 2020). However, reducing dietary phosphorus without exacerbating protein-energy wasting can be challenging (Gutiérrez and Wolf, 2010). Protein-energy wasting, in turn, is known to increase the risk of mortality (Brown-Tortorici et al., 2022). Therefore, phosphate binders appeared to be more feasible clinical therapeutic options for ESRD patients with hyperphosphatemia. On the other hand, previous meta-analytical studies have highlighted the risks of hypercalcemia and vascular calcification associated with the use of CBPBs despite their promising phosphorus-lowering effects in patients on dialysis (Liu et al., 2014; Yang et al., 2022). Accordingly, the latest version of the KDIGO treatment guidelines emphasize the need for dosage limitations in the use of CBPBs (Ruospo et al., 2018; Chaiyakittisopon et al., 2021). Although NCBPBs might be another clinical option, they are linked to undesirable side effects. For instance, lanthanum, sevelamer, and iron, the three common components of NCBPBs, are likely to cause nausea, constipation, and diarrhea, respectively (Palmer et al., 2016).

Cuttlebone has long been used in traditional Chinese medicine as an antacid (Chien et al., 2015). Modern research has shown that cuttlebone has potential therapeutic effects against gastrointestinal diseases (Huang et al., 2017) as well as anticancer and antioxidant properties (Kang et al., 2015). In patients with high blood phosphorus levels, cuttlebone has been used as a phosphorus-binding agent due to its high contents of calcium and other trace elements (Baek et al., 2022), which can bind to phosphorus. Additionally, because cuttlebone is not immediately absorbed after ingestion, it does not need to be taken with meals and has less adverse impact on appetite compared with other phosphorus-lowering agents (Xu et al., 2020).

The main finding of the current systematic review of nine RCTs was the significantly lower risk of phosphate hyperphosphatemia in patients receiving cuttlebone treatment compared to that in the control group. In addition, a dose-dependent effect on hyperphosphatemia has been noted with an efficacy for reducing serum phosphate levels being observed at medium-to-large dosages (i.e., 1.5–3 g TID and 5 g TID) but not at a low dosage (i.e., ≤1.5 g). Furthermore, cuttlebone exhibited beneficial effects after both short-term (i.e., 1–2 months) and long-term (3–6 months) administrations. By comparison, although lanthanum carbonate (i.e., a NCBPB) can also attain dose-related reductions in serum phosphorus after short- or long-term treatment (Guo et al., 2013), it is absorbed in the gut and has a biliary route of excretion. Besides, lanthanum has raised concern about tissue deposition (Lacour et al., 2005). Similarly, long-term and high-dose administration of CBPB has been linked to cardiovascular calcification and an increased risk of mortality (Jamal et al., 2013). Accordingly, the KDIGO guidelines recommend a limitation of the use of CBPBs in patients with high serum phosphate levels (Kidney Disease: Improving Global Outcomes CKDMBDUWG, 2011).

Regarding the therapeutic benefits of CBPBs and NCBPBs in this clinical setting, the latter are calcium-free and not associated with calcium-related complications. Nevertheless, they are more costly than the former (Rizk et al., 2016; Habbous et al., 2018; Chaiyakittisopon et al., 2021; Wang et al., 2022). In addition, one meta-analysis including fifty-one trials showed a nonsignificant reduction in mortality associated with the use of sevelamer (i.e., a NCBPB) despite significantly lower hospitalization rates and hypercalcemia compared with CBPBs. Besides, lanthanum and iron-based binders did not show superiority over CBPBs for any clinically relevant outcomes including cardiac events and fractures (Habbous et al., 2017). Therefore, the optimal phosphate binders for hyperphosphatemia management in chronic kidney disease (CKD) remain unclear. The current meta-analysis demonstrated the beneficial effect of cuttlebone on the reduction of serum phosphate regardless of the type of regimens (i.e., calcium-based vs. non-calcium-based) used in the control group. Another previous systematic review has shown that the use of sevelamer and cuttlebone as a combined treatment for hyperphosphatemia resulted in superior outcomes, including reductions in serum phosphate levels, calcium-phosphorus product, low-density lipoprotein cholesterol, and iPTH compared to those associated with monotherapy with either agent (Meng and Fu, 2015).

Excessive consumption of calcium supplements may lead to hypercalcemia. A high serum calcium-phosphorus product, defined as the product of serum calcium and phosphorus levels, can result in ectopic deposition of calcium phosphate crystals in vital organs such as the kidneys, lungs, and heart. This may lead to organ dysfunction and damage, thereby increasing the risks of morbidity and mortality (Thongprayoon et al., 1995). An elevated level of intact parathyroid hormone (iPTH), as in hyperparathyroidism, is another cause of increased levels of serum minerals, including calcium, phosphorus, and calcium-phosphorus product (Zhou X. et al., 2021). Regarding the concern about a possible increase in calcium levels associated with cuttlebone use, we found no significant difference in circulating calcium concentrations between patients treated with cuttlebone and those receiving NCBPBs or CBPBs. Furthermore, the current study demonstrated lower circulating levels of calcium-phosphorus product and iPTH in the cuttlebone group compared to those in the control group. On the other hand, our results showed that the risks of cardiovascular complications related to CBPBs and NCBPBs including vascular calcification, arteriosclerosis, and other cardiovascular diseases (Elder and Center, 2017) as well as gastrointestinal complications (Patel et al., 2016) were comparable to those associated with the use of cuttlebone in patients with end-stage renal disease. Taken together, our systematic review demonstrated the safety of cuttlebone and showed no difference in the risks of cardiovascular complications, constipation, gastrointestinal discomfort, and blood calcium elevation between those with and those without cuttlebone treatment.

Based on the type of dialysis (i.e., hemodialysis vs. peritoneal dialysis), our subgroup analysis revealed a more favorable response of cuttlebone in patients receiving hemodialysis than in those undergoing peritoneal dialysis. In concert with our finding, previous clinical trials have also shown a superior efficacy of hemodialysis compared to that of peritoneal dialysis against hyperphosphatemia (Kuhlmann, 2010; Evenepoel et al., 2016).

This study had several limitations. First, most studies failed to fully explain their randomization, allocation concealment, or blinding methods. Our overall risk of bias assessment that indicated “some concern” for bias in five studies and a high risk of bias in the other four trials may raise concerns regarding the reliability and validity of our findings, for which cautious interpretations of the overall conclusions drawn from our analysis are warranted. Second, the limited sample size in our meta-analysis was insufficient to advocate cuttlebone as equivalent or superior to established phosphate-lowering agents such as CBPBs and NCBPBs. Third, our inclusion of only Asian participants due to the therapeutic use of cuttlebone only in traditional Chinese medicine could not rule out the possibility of idiosyncratic side-effects in other ethnic groups. Therefore, besides a judicious interpretation of our findings, further large-scale studies are needed to address these issues. Fourth, the dosage range of cuttlebone in the intervention arm exhibited significant heterogeneity, precluding the ability to make direct dose-response comparisons. Furthermore, the control arm lacked standardized dosing regimens for the pharmacologic agents employed.

## 5 Conclusion

The results of the current meta-analysis suggest that cuttlebone may be a clinically efficient treatment for hyperphosphatemia comparable to conventional phosphate binders. The effectiveness of cuttlebone depended on the dosage, and it showed positive results in both short and long-term durations for reducing circulating phosphate levels. Our results may provide a foundation for researchers to investigate the feasibility of using cuttlebone as a potentially promising and cost-effective intervention for the treatment of hyperphosphatemia in patients with renal failure. Nevertheless, since the current study included a limited number of patients, additional large-scale clinical trials with more objective research designs are necessary to corroborate the findings of this study.

## Data Availability

The original contributions presented in the study are included in the article/Supplementary Material, further inquiries can be directed to the corresponding authors.
