# Supplementary material for: The efficacy and safety of cuttlebone for lowering serum phosphate in patients with end-stage renal disease: a meta-analysis of randomized controlled trials
Source: Front Pharmacol. 2023 Jul 24;14:1206366. doi: 10.3389/fphar.2023.1206366 (PMC10404851; doi:10.3389/fphar.2023.1206366)

**Supplemental Table 1.** Search strategies for Medline

| Database | # | Search syntax |
| --- | --- | --- |
| **MEDLINE (Ovid)** | 1 | (“cuttlebone” or “Hai-Piao-Xiao” or “cuttlefish bone” or “Sepiella maindroni de Rochebrune” or “Octopus japonicus” or “Sepiae Endoconcha”).mp |
|  | 2 | (“renal failure” or “end stage renal disease” or “dialysis” or “hemodialysis” or “peritoneal dialysis” or “kidney failure” or “renal insufficiency”).mp |
|  | 3 | exp "Kidney Failure, Chronic "/ OR exp "Renal Dialysis "/ OR exp "Peritoneal Dialysis"/ OR exp "Renal Insufficiency"/ OR exp “Kidney Failure, Chronic”/ |
|  | 4 | (“hyperphosphatemia” or “Serum phosphate” or “phosphorus”).mp |
|  | 5 | exp "Hyperphosphatemia"/ OR exp "Phosphates"/ |
|  | 6 | (1) AND (2 OR 3) AND (4 OR 5) |
|  | 7 | 6 AND (randomized controlled trial.pt. or controlled clinical trial.pt. or randomi*ed.ab. or placebo.ab. or drug therapy.fs. or randomly.ab. or trial.ab. or groups.ab. not (exp animals/ not humans.sh.)) |

**Supplemental Table 2:** Side effects of cuttlebone

|  | N^a^ | constipation | Gastrointestinal discomfort | hypercalcemia |
| --- | --- | --- | --- | --- |
| Cheng2018 | 60 | 1 | 0 | 0 |
| Lee 2012 | 30 | 2 | 1 | 0 |
| Liao 2019 | 32 | 1 | 1 | 0 |
| Liu 2005 | 25 | 3 | 0 | 0 |
| Tang 2016 | 40 | 0 | 0 | 0 |

^a^Intervention group

**Supplemental Figure 1.** Flowchart that illustrates the step-by-step methodology employed in the current meta-analysis. PICO: population, intervention, control, outcomes.


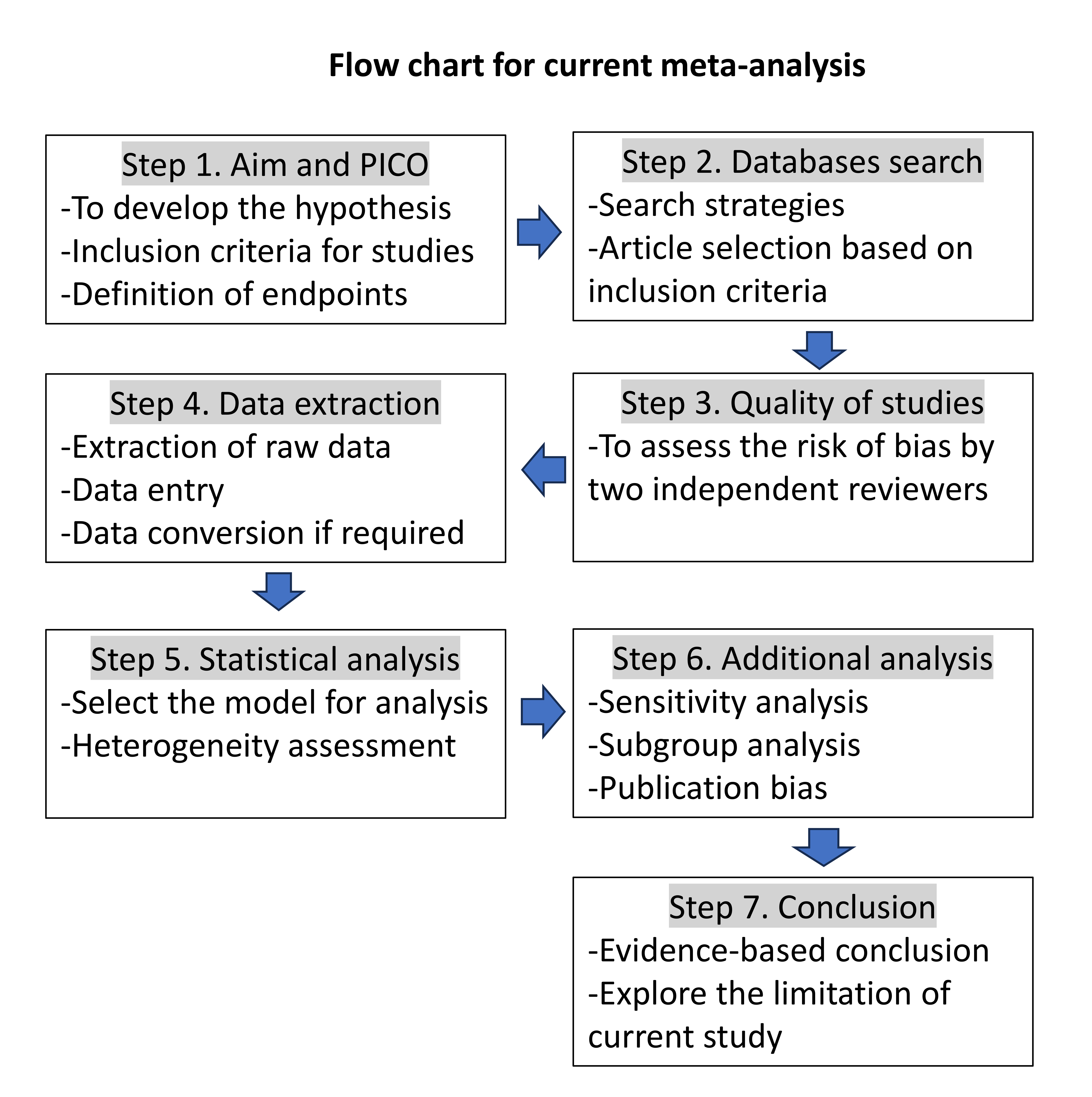


**Supplemental Figure 2.** Funnel plot showing a potential low risk of publication bias regarding the impact of cuttlebone on serum phosphate


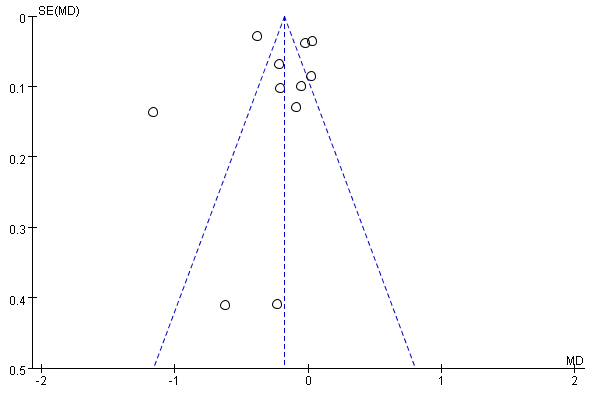


**Supplemental Figure 3.** Forest plot showing the beneficial effect of cuttlebone in lowing serum phosphate regardless of the type of regimens (Ca-based vs. non-Ca-based) used in the control group.


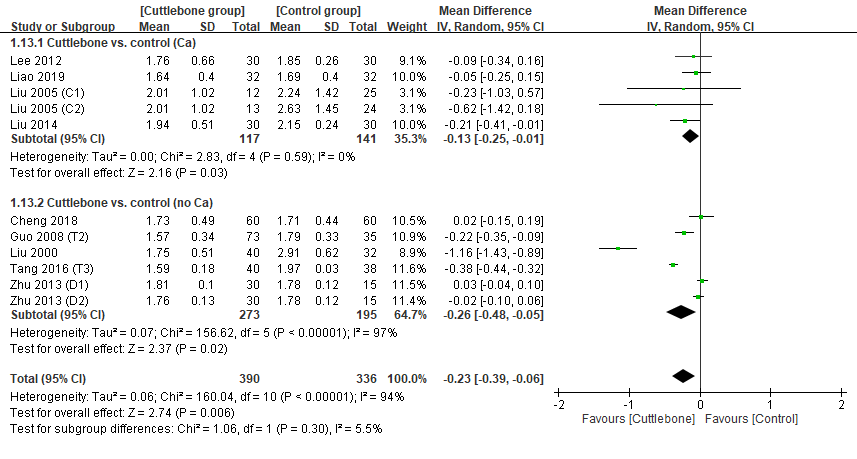


**Supplemental Figure 4.** Forest plot showing the beneficial effect of cuttlebone in lowing serum phosphate in patients receiving hemodialysis (HD), but not in those receiving peritoneal dialysis (PD).


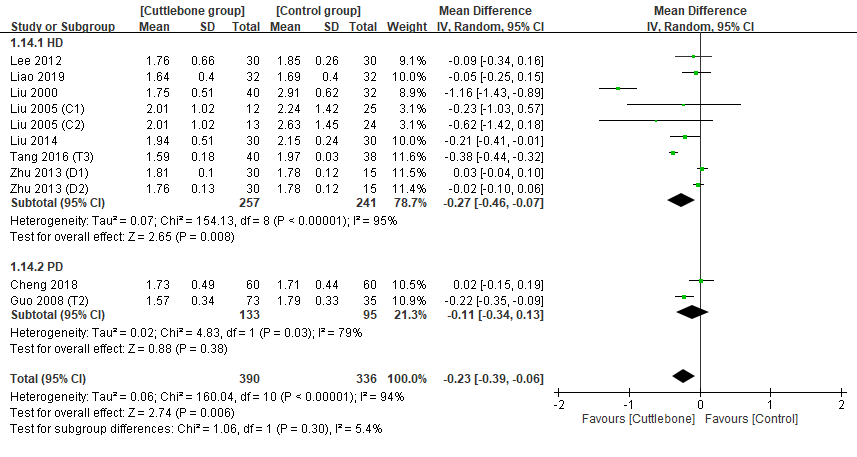


**Supplemental Figure 5.** Funnel plot showing a low risk of publication bias regarding the impact of cuttlebone on serum calcium.


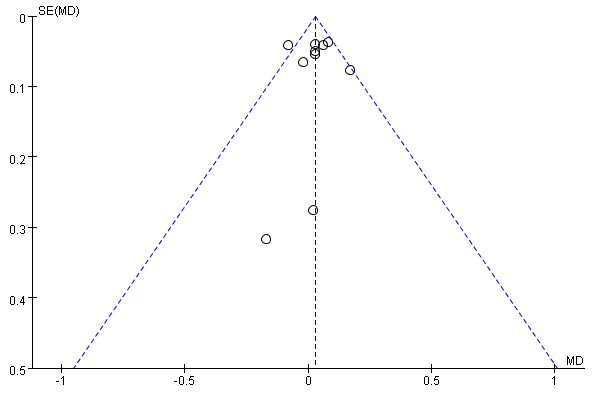


**Supplemental Figure 6.** Funnel plot showing a potential low risk of publication bias regarding the impact of cuttlebone on intact parathyroid hormone (iPTH) levels


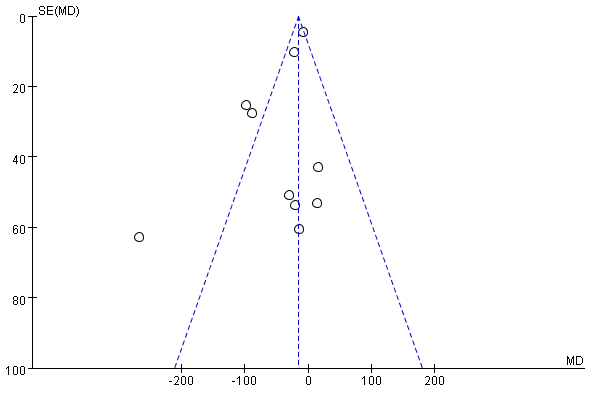

Supplement: Supplementary file 1 [file DataSheet1.docx]
